# Expression of activating receptors on natural killer cells from AIDS-related lymphoma patients

**DOI:** 10.1186/1742-6405-11-38

**Published:** 2014-11-24

**Authors:** Delphine Mercier-Bataille, Carole Sanchez, Céline Baier, Thérèse Le Treut, Nicolas Mounier, Saadia Mokhtari, Daniel Olive, Karine Baumstarck, Gérard Sébahoun, Caroline Besson, Régis T Costello

**Affiliations:** Université Aix-Marseille, Hôpital Nord, Laboratoire d’Hématologie, Chemin des Bourrely, 13915 Marseille Cedex 20, France; Technologies Avancées pour la Génomique et la Clinique (TAGC)/unité INSERM U1090, route de Luminy, 13008 Marseille, France; Service d’Oncologie-Hématologie, CHU de Nice, Hôpital Larchet, 151 Rte Saint Antoine Ginestiere, BP 7906202 Nice Cedex 3, France; Université Aix-Marseille, Hôpital Nord, Service des maladies infectieuses, Chemin des Bourrely, 13915 Marseille Cedex 20, France; Laboratoire d’immunologie des tumeurs, Institut Paoli-Calmette, bd Leï Roure, 13008 Marseille, France; Université Aix-Marseille, Faculté de La Timone, Unité d’Aide Méthodologique, 27 Boulevard Jean Moulin, 13005 Marseille, France; Service d’hématologie et immunologie clinique, CHU Bicêtre, 78 rue du Gal Leclerc, 94275 Le Kremlin-Bicêtre, France; Université Aix-Marseille, Hôpital La Conception, Service d’Hématologie et Thérapie Cellulaire, 147 Boulevard Baille, 13005 Marseille, France

**Keywords:** Natural killer cells, Natural cytotoxicity receptors, HIV-1, Lymphoma, Antitumor immune response

## Abstract

**Background:**

Abnormal NK phenotype and cytotoxic functions have been described in acute myeloid leukemia, chronic lymphocytic leukemia, myeloma and myelodysplastic syndromes. Defective NK cytotoxicity is due to decreased expression of the Natural Cytotoxicity Receptors (NCRs), 2B4/CD244/p38, or NKG2D. This prompted us to test the expression of these molecules on circulating NK cells from patients with AIDS-related lymphomas (RL) in comparison with HIV + patients without lymphoma, healthy subjects and HIV-negative patients with lymphoma.

**Methods:**

Blood samples were analyzed by flow cytometry for NCRs, 2B4/CD244/p38 and NKG2D expression on NK cells defined as CD3-/CD56+ lymphocytes. We also analyzed by quantitative PCR specific RNA for NKp30/NCR3 and NKp46/NCR1.

**Results:**

We could not detect any defect in NKp46/NCR1 expression between all groups. NKp44/NCR2, NKp30/NCR3 and NKG2D had lower expression in AIDS-RL in comparison with HIV + patients without lymphoma when compared to patients with similar (>0.3 G/L) CD4+ lymphocyte levels. Expression of 2B4/CD244/p38 was lower in AIDS-RL than in HIV-negative lymphoma. Comparison of specific NKp30/NCR3 and NKp46/NCR1 RNA showed increased steady state levels, despite decreased surface expression for NKp30/NCR3, suggesting abnormal post-transcriptional regulatory mechanisms.

**Conclusions:**

We show a more pronounced defect in NK activating molecule when HIV infection is associated with lymphoma than when only one condition (HIV positivity or lymphoma) is present. Defective NK phenotype, in addition to CD4+ depletion and dysfunction, may participate to the increased incidence of lymphoma in HIV patients.

## Background

Advances in lymphoma treatment prolong progression-free survival. Nonetheless, many patients relapse. Deficient cytotoxic functions of natural killer (NK) cells [1], which can be infected by HIV [2], may participate in the failure to cure AIDS-related lymphomas (AIDS-RL). Engagement of inhibitory receptors by human leukocyte antigen (HLA)-class-I molecules inhibits NK cytotoxicity. Thus, according to the “missing self hypothesis”, absent or deficient expression of HLA-class-I molecule activates NK if an additional activating signal is delivered by the natural cytotoxicity receptors (NCR) NKp30/NCR3, NKp44/NCR2 or NKp46/NCR1, 2B4/CD244/p38 and NKG2D. Deficient NK functions interfere with the anti-tumor response: 1) during treatment, via decreased efficiency of anti-CD20 antibody-driven cell cytotoxicity (ADCC) [3–5] 2) during the complete remission phase by favoring residual HLA-class-I negative lymphoma cells to escape from NK-mediated immunity [6]. Abnormal NK functions have been described in hematological malignancies such as acute myeloid leukemia, chronic lymphocytic leukemia, myeloma and myelodysplastic syndromes [7–10]. Of note, down-regulation of NCRs is associated with HIV infection [11]. We compared the NK cell surface activating molecules expression between patients with AIDS-RL, HIV-positive patients without lymphoma, lymphoma patients not infected by the HIV, and healthy subjects.

## Results

### Population characteristics

Among the 31 AIDS-RL (mean age: 43 ± 8 years) of the study, 20 had CD4+ lymphocytes <300/mm^3^ (mean 133 ± 70/mm^3^), 11 had CD4+ lymphocytes >300/mm^3^ (mean 630 ± 260/mm^3^). Only 2 patients (6.4%) were not treated by highly active antiretroviral therapy (HAART) at study inclusion. The control cohort included 56 HIV-positive patients without lymphoma (mean age: 44 ± 9 years) selected to be matched for the CD4+ lymphocyte count with the AIDS-RL: among this population, 9 patients [16%] were not treated by HAART. Two groups were designed: HIV patients with <300 CD4+ lymphocytes/mm^3^ (n = 12; mean age 44 ± 8.4 years, 1 patient without HAART) and HIV patients with >300 CD4+ lymphocytes/mm^3^ (n = 44; mean age 44 ± 9.8 years patients without HAART). Two other control cohorts of 33 HIV-negative lymphoma patients (mean age: 62 ± 14 years) and 19 healthy subjects (HS, mean age: 41 ± 16 years) were included.

### Lymphoid cells repartition (Figure 1– Panel A)

There was no significant difference in total lymphocytes and T-lymphocytes count among the different groups (p > 0.05). Lymphoma groups had more B lymphocytes (1700 ± 7000/mm^3^) than HIV (200 ± 200/mm^3^) and HS (300 ± 100/mm^3^) because of lymphoma circulating cells. Total NK cells counts in AIDS-RL with <300 CD4+ lymphocytes/mm^3^ and HIV + patients without lymphoma but with <300 CD4+ lymphocytes/mm^3^ were lower than in the other groups (mean 41 ± 35/mm^3^ for AIDS-RL <300 CD4+ lymphocytes/mm^3^ and 51 ± 52/mm^3^ for HIV + without lymphoma with <300 CD4+ lymphocytes/mm^3^*vs* 137 ± 257/mm^3^ for AIDS-RL with >300 CD4+ lymphocytes/mm^3^, 131 ± 148/mm^3^ for HIV + with >300 CD4+ lymphocytes/mm^3^, 276 ± 510/mm^3^ for HIV-negative lymphoma patients and 150 ± 70/mm^3^ for HS (p = 0.04 and 0.02).

**Figure 1:**
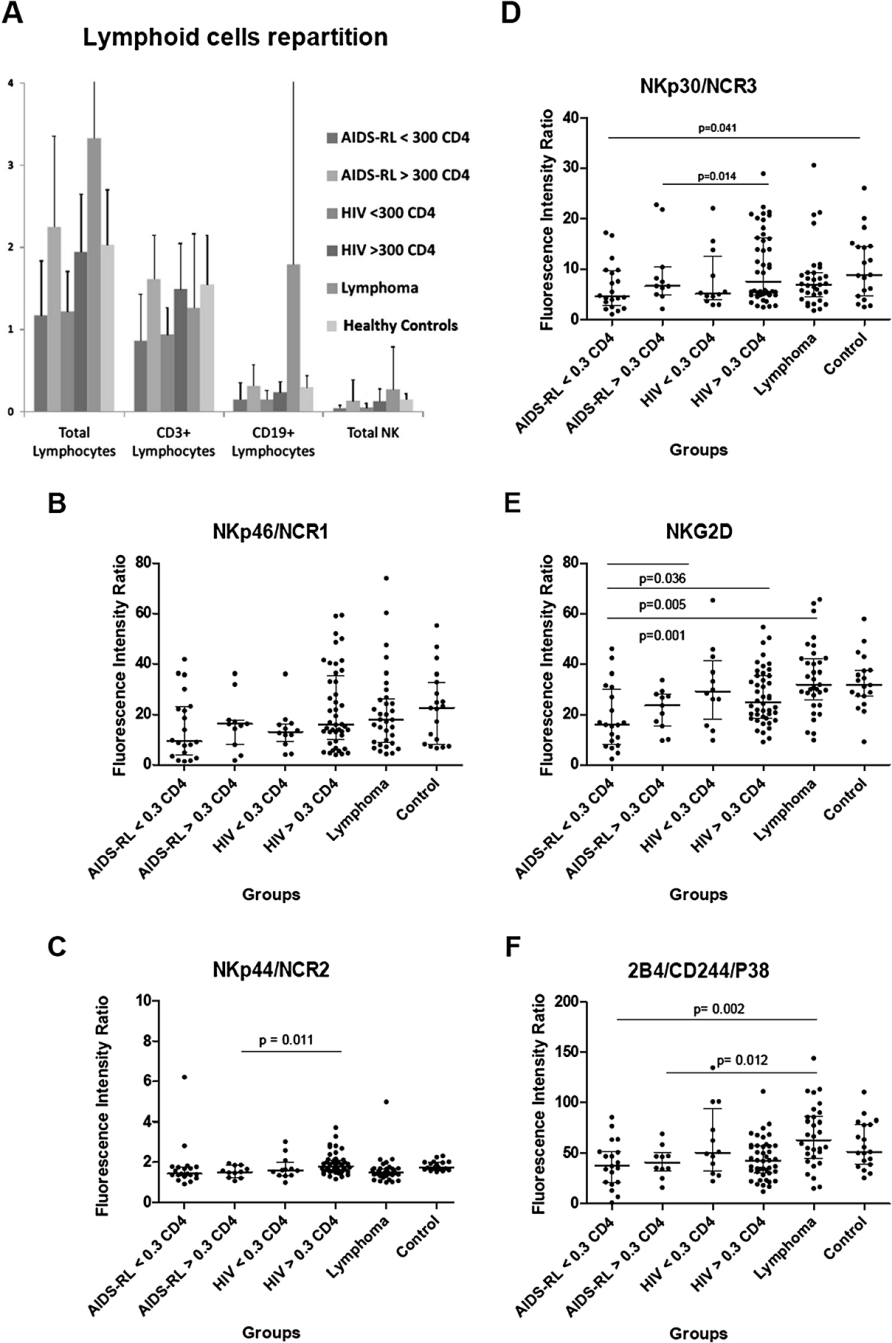
**Flow cytometry analysis of whole PBMC population (panel A) and of NK cells (panels B to F).** Results are expressed as absolute numbers of cells per volume unit, i.e. Giga/Liter in panel A. Results are expressed as mean fluorescence intensity ratio (in comparison with isotype controls, *cf.* Material and Methods). When significant, statistical results are indicated with the corresponding p-value. The number of analyzed patients was: AIDS-RL/CD4 < 300/mm3 = 20, AIDS-RL/CD4 > 300/mm3 = 11, HIV + <300 CD4/mm3 = 12, HIV+ > 400 CD4/mm3 = 44, non AIDS-RL lymphoma = 33, control HS = 19.

### NK activating receptors expression (Figure 1-Panel B, C, D, E, F)

No difference in NKp46/NCR1 expression was observed (Figure 1, panel B, p > 0.05, NS). The analysis of NKp44/NCR2 expression, an activating receptor only expressed on activated NKs (Figure 1, panel C) showed no difference between AIDS-RL and HIV-negative lymphoma patients (p > 0.05, NS) and between AIDS-RL and HIV patients without lymphoma but with <300 CD4/mm^3^ patients (p > 0.05, NS). However, AIDS-RL with >300 CD4+ lymphocytes/mm^3^ expressed lower NKp44/NCR2 than HIV + patients without lymphoma with >300 CD4+ lymphocytes/mm^3^ (MFIr median (M) = 1.52; interquartile range (IQR)[1.24-1.850] vs M = 1.80 IQR[1.540-2.110], p = 0.011) and than HS (M = 1.75 IQR[1.650-2.000], p = 0.006). In spite of a very high NKp44/NCR2 expression by one HIV + lymphoma with <300 CD4+ lymphocytes/mm^3^ (MFIr =6.21), this group tended to express lower NKp44/NCR2 than HIV + patients with >300 CD4+ lymphocytes/mm^3^ (M = 1.42 IQR[1.320-1.765] vs 1.80[1.543-2.115], p = 0.06). HIV-negative lymphoma patients expressed more NKp44/NCR2 than HIV + patients without lymphoma and >300 CD4+ lymphocytes/mm^3^ (M = 1.48 IQR[1.330-1.690] *vs* 1.80[1.543-2.115], p = 0.02) or HS (M = 1.75[1.650-2.000], p = 0.002). There was no difference in NKp30/NCR3 expression (Figure 1, panel D) between AIDS-RL and HIV-negative lymphoma patients (p > 0.05, NS) or between AIDS-RL and HIV + patients without lymphoma and <300 CD4+ lymphocytes/mm^3^ (p > 0.05, NS). NKp30/NCR3 expression was lower in AIDS-RL with >300 CD4+ lymphocytes/mm^3^ than in HIV + patients without lymphoma and >300 CD4+ lymphocytes/mm^3^ (M = 4.555 IQR[2.693-9.645] *vs* 7.525[4.853-16.19], p = 0.014) or HS (M = 8.86 IQR[4.630-14.56], p = 0.041).

Regarding NKG2D (Figure 1, panel E), AIDS-RL patients with <300 CD4+ lymphocytes/mm^3^ had a lower expression of NKG2D than AIDS-RL patients with >300 CD4+ lymphocytes/mm^3^ (M = 15.92 IQR[8.125-29.93] *vs* 29.18[18.18-41.32], p = 0.036), than HIV + patients without lymphoma but >300 CD4+ lymphocytes/mm^3^ (M = 24.92 IQR[18.68-35.45], p = 0.005), than HIV-negative lymphoma patients (M = 31.89 IQR[25.71-42.17], p = 0.001), or than HS (M = 31.78 IQR[27.40-37.50], p = 0.001). AIDS-RL with >300 CD4+ lymphocytes/mm^3^ had lower expression of NKG2D than HIV-negative lymphoma patients (M = 23.65 IQR[15.45-28.19] *vs* 31.89[25.71-42.17], p = 0.03), than HS (M = 31.78 IQR[27.40-37.50], p = 0.005) or HIV + patients without lymphoma but >300 CD4+ lymphocytes/mm^3^ (M = 24.92 IQR[18.68-35.45], p = 0.005).

We failed to detect 2B4/CD244/P38 expression difference (Figure 1, panel F) between AIDS-RL and HIV + patients without lymphoma (NS, p > 0.05). 2B4/CD244/P38 expression was lower in AIDS-RL with <300 CD4+ lymphocytes/mm^3^ than in HIV-negative lymphoma patients (M = 37.36 IQR[21.45-51.68] *vs* 63.43[45.16-86.86], p = 0.002) and than in HS (M = 51.64[38.93-78.76], p = 0.023). 2B4/CD244/P38 expression was lower in AIDS-RL with >300 CD4+ lymphocytes/mm^3^ than in HIV-negative lymphoma patients (M = 41.11 IQR[32.18-50.35] vs 63.43[45.16-86.86], p = 0.012).

### Quantitative RT-PCR of NKp30/NCR3 and NKp46/NCR1 (Figure 2)

**Figure 2:**
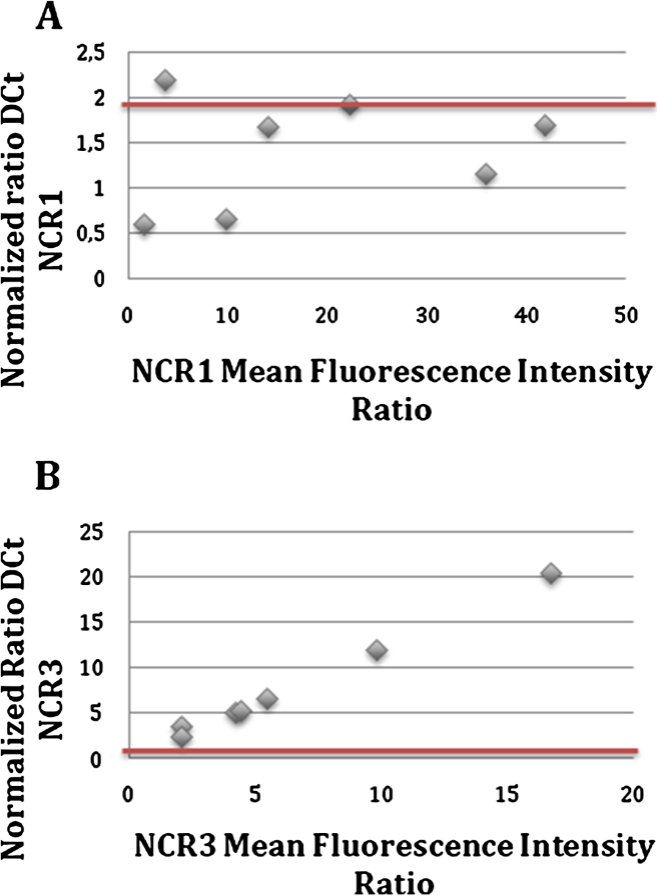
**RT-qPCR analysis of NKp46/NCR1 (panel A) and NKp30/NCR3 (panel B).** Values fewer than 2 correspond to an RNA expression not statistically superior to the gene control, value above 2 correspond to over-expressed RNA. Data correspond the RNA levels ratios between 7 AIDS-RL samples versus 32 HIV-positive patients without lymphoma.

In line with cell surface expression, AIDS-RL had no modification of NKp46/NCR1 specific RNA level (0.5 < normalized ratio <2 for the 6 of the 7 patients analyzed) compared with HIV + population without lymphoma (Panel A). We did not find any correlation between NKp46/NCR1 expression and CD4+ lymphocyte count. On the contrary, NKp30/NCR3 RNA (Panel B) was overexpressed in AIDS-RL patients (normalized ratio >2 for the 7 patients analyzed). No significant RNA level was detected for NKp44/NCR2, a results in line with normal physiology since this molecule is only expressed by stimulated NKs (data not shown).

## Discussion

The total NK cells count in patients with <300 CD4+ lymphocytes/mm^3^ was lower in AIDS-RL in comparison with the other groups, suggesting a poor prognosis as shown in low or high grade HIV-negative lymphomas [12, 13]. Low circulating counts concerned the NK CD56^bright^ and CD56^low^ subsets, while the ineffective CD56^negative^ subpopulation was elevated in AIDS-RL (data not shown). Regarding the NCR we found no difference in NKp46/NCR1 expression in the different groups but, in contrast, a significant decrease in both NKp44/NCR2 and NKp30/NCR3 was observed in AIDS-RL with >300 CD4/mm^3^ in comparison with HIV + patients with comparable CD4+ lymphocytes. Thus in moderately immune-suppressed patients the development of lymphoma is associated with low expression of two activating molecules. Regarding NKp44/NCR2, low levels should be of good prognosis since NK cells in HIV-controller patients do not up-regulate NKp44/NCR2 thus protecting uninfected CD4+ lymphocytes from inadequate NK killing [14]. Regarding the mechanism of NCR regulation, quantitative RT-PCR measured comparable NKp46/NCR1 levels in AIDS-RL and controls, suggesting an identical regulation at both transcriptional and post-transcriptional levels. However elevated level of NKp30/NCR3 specific RNA was detected in AIDS-RL in comparison with HIV patients without lymphoma, despite identical surface expression of NKp30/NCR3. This suggests that a post-transcriptional mechanism negatively interferes with NKp30/NCR3 RNA traduction or protein stability, leading to identical surface expression despite higher specific RNA levels. Regarding NKG2D, we observed a gradient of expression from lower level (AIDS-RL <300 CD4/mm^3^) to HS/HIV-negative lymphoma patients, with intermediary levels for AIDS-RL with >0.3 G/L CD4+ lymphocytes followed by HIV + patients without lymphoma. The NKG2D ligands MICA/MICB/ULBP are stress molecules expressed on tumor cells, and secreted at high levels in HIV patients, leading to down-regulated NKG2D expression on NK and impaired anti-lymphoma cytotoxicity [15]. Expression of 2B4/CD244/p38 was lower in AIDS-RL than in HIV-negative lymphoma patients. The 2B4/CD244/p38 ligand is the CD48 molecule [16, 17] which is expressed on B normal and neoplastic lymphocyte and is drastically up-regulated by Ebstein-Barr virus (EBV) infection. The down-regulation of 2B4/CD244/p38 could thus impair the cytotoxicity against EBV-positive B-cell lymphomas.

## Conclusion

The AIDS-RL patients had decreased levels of 2 out of 3 NCRs, of NKG2D and of 2B4/CD244/p38. The most significant difference concerned NKG2D, which expression was significantly decreased regarding both HIV patients without lymphoma, non-HIV lymphoma patients and HS. This specific abnormality is of great interest since lymphoma cells express the stress ligands MICA/B and ULBP, but may escape to NK cytotoxicity due to impaired NKG2D expression. Of note, HAART was not sufficient to restore a normal phenotype since most of our patients were already treated at the time of NK phenotype analysis, with NK abnormalities also detected in patient with CD4+ lymphocytes >300/mm^3^. Since defects in NK immune surveillance may also impair the anti-infectious immunity, they could also partly explain the susceptibility to infection of HIV patients during chemotherapy, even in patients with high CD4+ T-lymphocytes levels. Altogether our data suggest than immune intervention aiming at NK cell function restoration could be of interest in AIDS-RL patients.

## Material and methods

### Study design

According to previous data [8], we hypothesized that 75% ±10% of AIDS-RL patients and 15% ±10% of HIV patients without lymphoma had low NCR expression (NCR^dull^). In order to show a statistically significant difference between the 2 groups with a risk α = 5% and β = 75%, we included in our study 31 AIDS-RL patients and 56 HIV positive patients without lymphoma.

### Patients

From July 2006 to June 2011 patients from Marseille, Nice and Paris were included in first line of therapy. Inclusion criteria were the co-existence of HIV infection with biopsy-proven lymphoma. The 56 HIV positive patients without lymphoma were recruited from Service des Maladies Infectieuses (Hôpital Nord, Marseille). According to Helsinki declaration, patients were informed and signed a consent form. Biological samples were collected at diagnosis time, before lymphoma treatment. Additional comparison of our data was also performed with 33 non-HIV patients with lymphoma and 19 healthy subjects (HS).

This study was approved by the Comité de Protection des Personnes (CPP) Aix-Marseille II.

### Blood samples and cell separation

Blood samples were collected on EDTA and analyzed by flow cytometry. Dry pellets of PBMC were frozen at -80°C for subsequent quantitative RT-PCR analysis.

### qRT-PCR analysis

qRT-PCR analysis concerned 7 AIDS-RL patients and 32 HIV-positive patients without lymphoma. qRT-PCR analysis was performed with the Applied Biosystems 7900HT Fast Real-Time PCR system using Taqman detection. Total RNA was isolated using TRIzol reagent (Invitrogen Life Technologies). Capture of fluorescence was recorded on the ABI Prism 7900HT scanner and the Ct (threshold cycle) was calculated for each assay (Sequence Detection System Software 2.3, Applied Biosystems). We used GAPDH as endogenous control (ΔCt = Ct target gene - Ct GAPDH). GAPDH TaqMan Gene Expression assays were from Applied Biosystems. Since the NCR expression is almost exclusively restricted to NK, the PCR was performed on the whole PBMC population, but the values were adjusted to the percentage of NK present in each sample. We compared ΔCt with the mean of VIH ΔCt using a ratio (ΔCt HIV + Lymphoma/ΔCt HIV), considering that a ratio >2 corresponded to RNA overexpression.

### Phenotypic analysis

Flow cytometry was performed on an Epics XL^R^ flow cytometer (Beckman Coulter). The NK cells were defined as CD3-/CD56+/CD16+. The following mAbs were used (Beckman-Coulter, Marseille, France): anti-CD3^FITC^ (UCHT1), anti-CD56^PC5^ (N901-NKH1), anti-NCR1/NKp46^PE^ (BAB281), anti-NCR2/^NKp44PE^ (Z231), anti-NCR3/NKp30^PE^ (Z25), anti-NKG2D-PE (ON72), anti-P38 (C1.7), anti-IgG1^-FITC^, anti-CD19^-PC5^ (J4.119), CD4^-PE^/CD8^-ECD^/CD3^-PC5^, anti-CD3^-FITC^/CD16^-PE^ (UCHT1/3G8). All our results were expressed as the mean fluorescent intensity ratio molecule of interest/isotypic control (MFIr).

### Statistical analysis

Data were compared between the 4 groups using a nonparametric Kruskal-Wallis test; post hoc tests for multiple comparisons were performed when the test was significant (macro Marta Garcia-Granero [07/2008] for SPSS). The statistical analyses were performed using the SPSS software package, version 17.0 (SPSS Inc., Chicago, IL, USA). All tests were two-sided. Statistical significance was defined as p <0.05.

## Abbreviations

NK: Natural killer
NCR: Natural cytotoxicity receptors
AIDS-RL: AIDS-related lymphoma
HS: Healthy sublects
MFI: Mean fluorescent intensity
PBMC: Peripheral blood mononucleated cells
PCR: Polymerase chain reaction.

## References

[1] Altfeld M, Fadda L, Frleta D, Bhardwaj N (2011). DCs and NK cells: critical effectors in the immune response to HIV-1. Nat Rev Immunol.

[2] Valentin A, Rosati M, Patenaude DJ, Hatzakis A, Kostrikis LG, Lazanas M, Wyvill KM, Yarchoan R, Pavlakis GN (2002). Persistent HIV-1 infection of natural killer cells in patients receiving highly active antiretroviral therapy. Proc Natl Acad Sci U S A.

[3] Ahmad A, Yao XA, Tanner JE, Cohen E, Menezes J (1994). Surface expression of the HIV-1 envelope proteins in env gene-transfected CD4-positive human T cell clones: characterization and killing by an antibody-dependent cellular cytotoxic mechanism. J Acquir Immune Defic Syndr.

[4] Forthal DN (1999). Cytotoxic T, lymphocyte precursors in persons with repeated exposure to human immunodeficiency virus. J Infect Dis.

[5] Forthal DN, Landucci G, Daar ES (2001). Antibody from patients with acute human immunodeficiency virus (HIV) infection inhibits primary strains of HIV type 1 in the presence of natural-killer effector cells. J Virol.

[6] Farnault L, Sanchez C, Baier C, Le Treut T, Costello RT (2012). Hematological malignancies escape from NK cell innate immune surveillance: mechanisms and therapeutic implications. Clin Dev Immunol.

[7] Costello RT, Sivori S, Marcenaro E, Lafage-Pochitaloff M, Mozziconacci MJ, Reviron D, Gastaut JA, Pende D, Olive D, Moretta A (2002). Defective expression and function of natural killer cell-triggering receptors in patients with acute myeloid leukemia. Blood.

[8] Fauriat C, Just-Landi S, Mallet F, Arnoulet C, Sainty D, Olive D, Costello RT (2007). Deficient expression of NCR in NK cells from acute myeloid leukemia: Evolution during leukemia treatment and impact of leukemia cells in NCRdull phenotype induction. Blood.

[9] Costello RT, Knoblauch B, Sanchez C, Mercier D, Le Treut T, Sébahoun G (2012). Expression of natural killer cell activating receptors in patients with chronic lymphocytic leukaemia. Immunology.

[10] Kiladjian JJ, Bourgeois E, Lobe I, Braun T, Visentin G, Bourhis JH, Fenaux P, Chouaib S, Caignard A (2006). Cytolytic function and survival of natural killer cells are severely altered in myelodysplastic syndromes. Leukemia.

[11] De Maria A, Fogli M, Costa P, Murdaca G, Puppo F, Mavilio D, Moretta A, Moretta L (2003). The impaired NK cell cytolytic function in viremic HIV-1 infection is associated with a reduced surface expression of natural cytotoxicity receptors (NKp46, NKp30 and NKp44). Eur J Immunol.

[12] Shafer D, Smith MR, Borghaei H, Millenson MM, Li T, Litwin S, Anad R, Al-Saleem T (2013). Low NK cell counts in peripheral blood are associated with inferior overall survival in patients with follicular lymphoma. Leuk Res.

[13] Plonquet A, Haioun C, Jais JP, Debard AL, Salles G, Bene MC, Feugier P, Rabian C, Casasnovas O, Labalette M, Kuhlein E, Farcet JP, Emile JF, Gisselbrecht C, Delfau-Larue MH, Groupe d'étude des lymphomes de l'adulte (2007). Peripheral blood natural killer cell count is associated with clinical outcome in patients with aaIPI 2–3 diffuse large B-cell lymphoma. Ann Oncol.

[14] Marras F, Nicco E, Bozzano F, Di Biagio A, Dentone C, Pontali E, Boni S, Setti M, Orofino G, Mantia E, Bartolacci V, Bisio F, Riva A, Biassoni R, Moretta L, De Maria A (2013). Natural killer cells in HIV controller patients express an activated effector phenotype and do not up-regulate NKp44 on IL-2 stimulation. Proc Natl Acad Sci U S A.

[15] Matusali G, Tchidjou HK, Pontrelli G, Bernardi S, D'Ettorre G, Vullo V, Buonomini AR, Andreoni M, Santoni A, Cerboni C, Doria M (2013). Soluble ligands for the NKG2D receptor are released during HIV-1 infection and impair NKG2D expression and cytotoxicity of NK cells. FASEB J.

[16] Brown MH, Boles K, van der Merwe PA, Kumar V, Mathew PA, Barclay AN (1998). 2B4, the natural killer and T cell immunoglobulin superfamily surface protein, is a ligand for CD48. J Exp Med.

[17] Latchman Y, McKay PF, Reiser H (1998). Identification of the 2B4 molecule as a counter-receptor for CD48. J Immunol.

